# Heat degradation of eukaryotic and bacterial DNA: an experimental model for paleomicrobiology

**DOI:** 10.1186/1756-0500-5-528

**Published:** 2012-09-25

**Authors:** Tung Nguyen-Hieu, Gérard Aboudharam, Michel Drancourt

**Affiliations:** 1 URMITE, UM63, CNRS 7278, IRD 198, Inserm 1095, Aix-Marseille Université, 27 boulevard Jean Moulin, Marseille, 13005, France; 2 Unité des Rickettsies, Faculté de Médecine, 27 Boulevard Jean Moulin, Marseille Cedex 05, 13385, France

**Keywords:** Ancient DNA, DNA degradation, Bacterial DNA, Eukaryotic DNA, *Mycobacterium*, Real-time PCR

## Abstract

**Background:**

Theoretical models suggest that DNA degradation would sharply limit the PCR-based detection of both eukaryotic and prokaryotic DNA within ancient specimens. However, the relative extent of decay of eukaryote and prokaryote DNA over time is a matter of debate. In this study, the murine macrophage cell line J774, alone or infected with *Mycobacterium smegmatis* bacteria, were killed after exposure to 90°C dry heat for intervals ranging from 1 to 48 h in order to compare eukaryotic cells, extracellular bacteria and intracellular bacteria. The sizes of the resulting mycobacterial *rpo*B and murine *rpb*2 homologous gene fragments were then determined by real-time PCR and fluorescent probing.

**Findings:**

The cycle threshold (Ct) values of PCR-amplified DNA fragments from J774 cells and the *M. smegmatis* negative controls (without heat exposure) varied from 26–33 for the J774 *rpb*2 gene fragments and from 24–29 for *M. smegmatis rpo*B fragments. After 90°C dry heat incubation for up to 48 h, the Ct values of test samples increased relative to those of the controls for each amplicon size. For each dry heat exposure time, the Ct values of the 146-149-bp fragments were lower than those of 746-747-bp fragments. During the 4- to 24-h dry heat incubation, the non-infected J774 cell DNA was degraded into 597-bp *rpb*2 fragments. After 48 h, however, only 450-bp *rpb*2 fragments of both non-infected and infected J774 cells could be amplified. In contrast, the 746-bp *rpo*B fragments of *M. smegmatis* DNA could be amplified after the 48-h dry heat exposure in all experiments. Infected and non-infected J774 cell DNA was degraded more rapidly than *M. smegmatis* DNA after dry heat exposure (ANOVA test, p < 0.05).

**Conclusion:**

In this study, mycobacterial DNA was more resistant to dry-heat stress than eukaryotic DNA. Therefore, the detection of large, experimental, ancient mycobacterial DNA fragments is a suitable approach for paleomicrobiological studies.

## Findings

### Introduction

The seminal demonstration that nuclear DNA could be cloned from a 2,400-year-old Egyptian mummy [1] founded molecular paleontology and paleomicrobiology [2,3]. However, the cumulative experience over the past few decades indicates that the detection of ancient DNA (aDNA) could be limited by DNA degradation influenced by pH and humidity of the burial site. Heat, ultraviolet rays and oxidative agents are also proposed to contribute to the alteration of the chemical nature of nucleic acid bases and the degradation of DNA in buried human and animal remains [4-8]. Based on theoretical models of DNA degradation [4,9,10], some authors have questioned the long-term stability of DNA and suspected that some paleomicrobiological detections may have resulted from the contamination of samples with modern DNA [5,11]. Studies have demonstrated aDNA to be degraded into < 150-bp nuclear and < 400-bp mitochondrial fragments [12,13]. Experimental models of DNA degradation have used purified DNA [14-16] because few studies have assessed the degradation of DNA within cells [17]. Moreover, these early studies used eukaryotic DNA. Indeed, the potential degradation of prokaryotic aDNA was only extrapolated from the data obtained from eukaryotic aDNA without experimental validation. Some authors have proposed, however, that in ancient, buried specimens, bacterial DNA may be more resistant to decay over time than human DNA [18,19]. In this study, we evaluated experimental DNA decay in both eukaryotic and prokaryotic cells.

#### Dry heat degradation of murine macrophage cell line J774 DNA and *M. smegmatis* DNA

**Figure 1:**
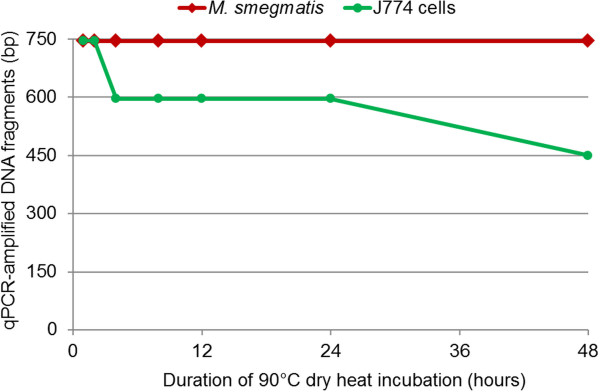
**Real-time PCR quantification of dry heat DNA degradation of *M. smegmatis *and J774 cells.**

One-hour incubation of *M. smegmatis* and J774 cells at 90°C resulted in cell death as indicated by the absence of subculture. All blank control PCR reactions were negative. The cycle threshold (Ct) values of PCR-amplified fragments from J774 DNA and *M. smegmatis* negative control DNA (without heat exposure) varied from 26–33 for J774 cell *rpb*2 fragments and 24–29 for *M. smegmatis rpo*B fragments. For test samples exposed to 90°C dry heat for intervals ranging from 1 to 48 h, the Ct values of PCR-amplified fragments were observed to increase relative to those of controls at each amplicon size tested. Furthermore, for each dry heat exposure time, the Ct values of the shorter 146-149-bp fragments were lower than those of the longer 746-747-bp fragments (Additional file 1: Table S1). During the 4- to 24-h dry heat exposures, J774 cell DNA degraded into 597-bp fragments. After 48 h of exposure, however, only 450-bp *rpb*2 fragments of J774 cell DNA could be amplified. In contrast, 746-bp *rpo*B fragments of *M. smegmatis* DNA could be amplified even after 48 h of dry heat exposure (Figure 1). The DNA degradation of J774 cells was more rapid than that of *M. smegmatis* through 1 – 48 – hour exposure to dry heat (ANOVA test, p < 10^-3^, Additional file 2: Table S2).

#### Dry heat degradation of *M. smegmatis*-infected J774 cell DNA

**Figure 2:**
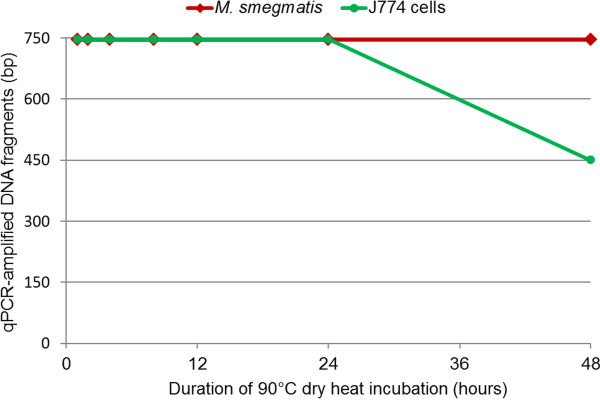
**Real-time PCR quantification of dry heat DNA degradation of *M. smegmatis *-infected J774 cells.**

In the experiments utilizing intracellular bacteria, all blank controls were negative. *M. smegmatis*-infected J774 cells not exposed to dry heat served as negative controls, and the Ct values of PCR-amplified DNA varied from 23 – 30 for J774 fragments and 27 – 28 for *M. smegmatis* fragments. After exposure to 90°C dry heat for durations ranging from 1 to 48 h, the Ct values of test samples were observed to increase relative to those of controls for each amplicon size. Additionally, for each dry heat exposure time, the Ct values of the shorter 146-149-bp fragments were lower than those of the longer 746-747-bp fragments (Additional file 3: Table S3). After 48 h of dry heat exposure, only the 450-bp fragments of infected J774 cell DNA could be amplified. In contrast, the 746-bp fragments of intracellular *M. smegmatis* DNA could be amplified following the same 48-h dry heat exposure (Figure 2). The DNA degradation of the infected-J774 cells was more rapid than that of intracellular *M. smegmatis* through 2 – 12 – hour and 48 – hour exposure to dry heat (ANOVA test, p < 0.05, Additional file 2: Table S2). There was no statistically significant difference in the degradation of J774 cell DNA, between *M. smegmatis*-infected and non-infected cells (ANOVA test, p > 0.05, Additional file 2: Table S3).

The data presented in this manuscript can be interpreted as both authentic and biologically relevant. All blank controls used in our PCR-based experiments were negative. Additionally, the control cells not exposed to dry heat yielded the expected results. Macrophages and mycobacteria were exposed to dry heat in parallel, and the *rpo*B gene of mycobacteria was assessed in parallel with its homolog, the *rpb*2 gene of murine macrophages. Reproducible results were observed across experiments performed in triplicate.

The dry heat used in this study has been used previously to assess experimental degradation of purified DNA [16,17] and is the most common environmental agent that may cause DNA damage in dead cells. Thus, our experiments utilizing dry heat exposure mimic an accelerated natural DNA degradation process. Furthermore, in this study, dry heat was applied to cellular DNA rather than purified DNA. We verified that these conditions killed mycobacteria. However, the experimental design of the study did not allow us to rule out the hypothesis that viable non-culturable mycobacteria persisted into infected macrophages [20], possibly interfering with macrophage DNA degradation. Additionally, all previously published experiments have used agarose gel electrophoresis to monitor DNA degradation [14-17]. This method of evaluation is imprecise, as it relies on the visual observation of smears and only provides estimates as to the extent of DNA degradation. In our study, we used real-time PCR in combination with TaqMan® fluorescent probes to accurately monitor the size of PCR-amplifiable fragments of mycobacterial and eukaryotic DNA following exposure to heat stress. *M. smegmatis* was chosen because it has a lipid-rich cell wall similar to that of *Mycobacterium tuberculosis*, a pathogen previously investigated in paleomicrobiological studies [3,18]. Furthermore, *M. smegmatis*-infected J774 cells were used as an experimental model with which to assess DNA degradation of obligate intracellular bacteria. This model of particular interest because *Rickettsia prowazekii* and *Mycobacterium leprae*, which are obligate intracellular bacteria, have been detected in ancient specimens [21-24]. Our studies assessing experimental and comparative degradation of cellular DNA in both bacteria and eukaryotic cells are the first in the literature.

**Figure 3:**
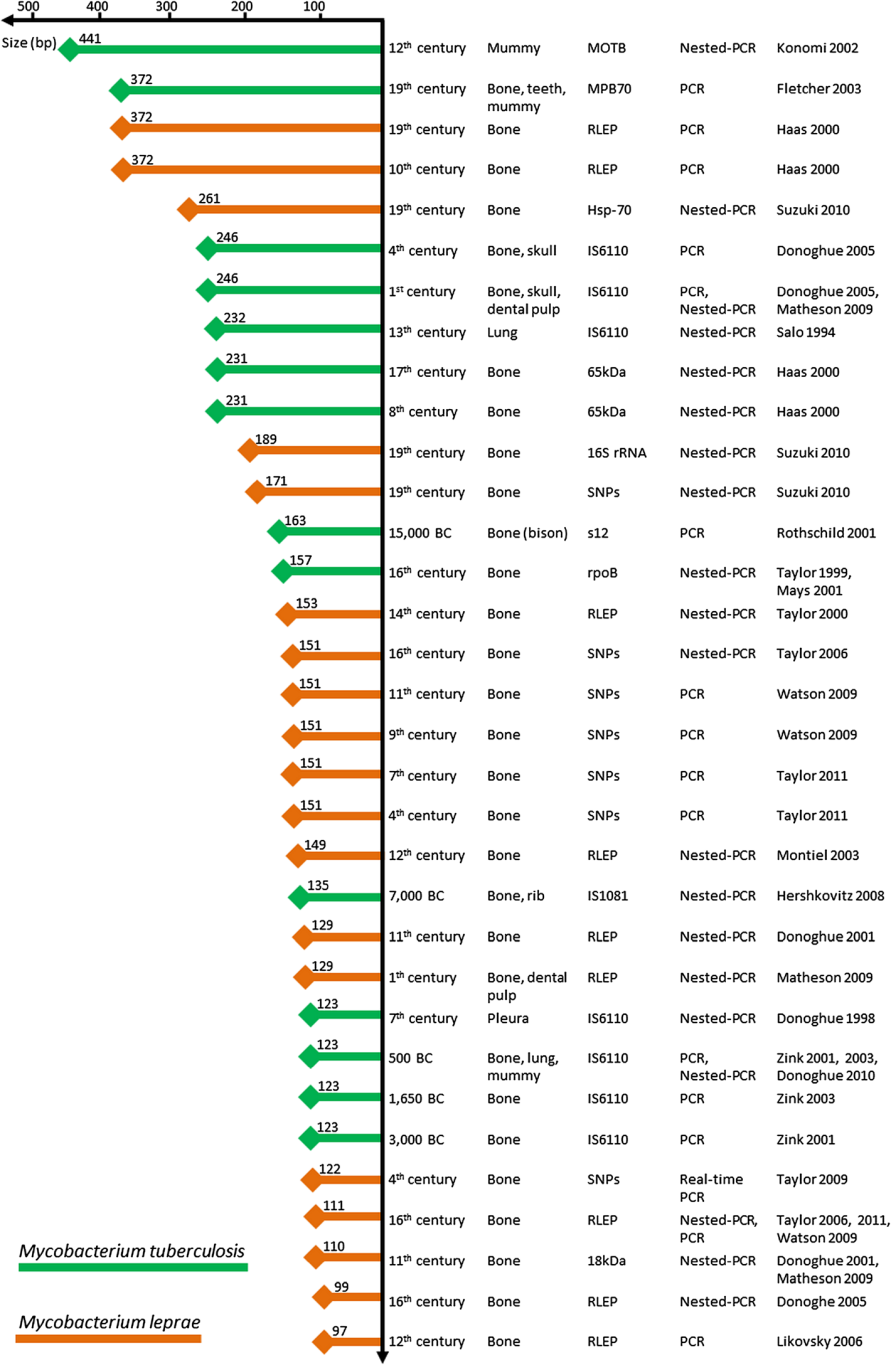
**Mycobacterial DNA fragments PCR-amplified from ancient specimens (References included in the Additional file****4****: Table S4).**

Several criteria have been published to authenticate aDNA-based data [2,11]. These include the absence of a positive control, negativity of negative controls, sequencing all PCR amplicons, amplification and sequencing of a second target, originality of the ancient sequences and reproducibility of results in at least two independent laboratories. The data presented here are consistent with previously published paleomicrobiological observations. DNA fragments exceeding 350-bp have been PCR-amplified from several ancient bacterial pathogens, including *M. tuberculosis* and *M. leprae*, from a number of ancient, buried individuals (Figure 3, Additional file 4: Table S4). Bone, mummified tissues (e.g. mummy, skull, lung, pleura, and rib) and dental pulp are often used for these types of paleomicrobiological investigations (Figure 3), although aDNA is more easily extracted from soft tissues than from calcified ones. The data reported herein cannot be extrapolated to other bacteria, nevertheless DNA fragments larger than 250-bp from infectious agents have been successfully PCR-amplified from ancient dental pulp specimens [24-31]. This finding supports the hypothesis that dental pulp is a good source of genetic material for DNA-based paleomicrobiological studies [32]. The published data indicate that the average eukaryotic DNA fragment size in these experiments does not correlate with the age of ancient specimens (Figure 3), suggesting that DNA degradation occurs relatively soon after cell death [4,12]. Indeed, 250-bp average fragments have been sequenced from both a 30,000-year-old permafrost sediment [33] as well as a 40,000-year-old cave bear bone [34]. Interestingly, most large DNA fragments recovered from permafrost sediment are comprised of mostly bacterial DNA, supporting the idea that bacterial DNA was better preserved than eukaryotic DNA in ancient specimens.

### Conclusions

Field observations, along with the experimental data presented here, suggest that mycobacterial DNA is more resistant than eukaryotic DNA to taphonomic degradation. Bacterial DNA fragmentation could be offset by DNA repair activities [35,36]. The measurement of carbon dioxide release from bacteria embedded in permafrost for 500,000 years found that bacterial metabolic activity ensured survival and DNA repair capacity [37]. Additionally, thick cell walls, like those of mycobacteria, could protect bacterial DNA from certain degrading agents [18,19]. The dogma that ancient bacterial DNA is fragmented to an extent where only targets shorter than 200-bp can be detected [38] is not supported by either the experimental data or the high-throughput pyrosequencing observations. Large bacterial DNA fragments can be detected from ancient buried specimens without enzymatic reparation [39].

### Methods

#### Culture of murine macrophage cell line J774 and *M. smegmatis*

The murine macrophage J774 (ATCC TIB 67) cells were cultured in GIBCO® 1X DMEM culture medium (Invitrogen, Carlsbad, USA) supplemented with 10% heat-decomplemented fetal calf serum (Seromed, Strasbourg, France) and 1% glutamine (Seromed) at 37°C with 5% CO_2_ for 3 days. *M. smegmatis* mc2 (ATCC 700084) was cultured in trypticase-soy-casein broth (European Pharmacopia IV, Strasbourg, France) supplemented with 0.5% Tween 80 (European Pharmacopia IV) at 37°C for 10 days. For the co-culture experiment, 1.8 mL of a 10^6^ mycobacteria/mL suspension were incubated with 15 mL of a 10^5^ J774 cells/mL suspension at 37°C under 5% CO_2_ atmosphere for 4 h. The infected J774 cell monolayer was then washed two times with 15 mL sterile phosphate buffered saline (PBS) before 15 mL fresh culture medium supplemented with 1% streptomycin (Panpharma, Fougères, France) was added for 2 h, eliminating any extracellular mycobacteria [40]. The infected-cell monolayer was then washed two times with 15 mL sterile PBS before 15 mL of fresh culture medium was added. The monolayer was incubated at 37°C with 5% CO_2_ for 24 h. Infection of the J774 cell layer was monitored by Ziehl-Neelsen staining. The viability of *M. smegmatis* mycobacteria and J774 cells after one-hour incubation at 90°C was assessed by subculture as described above.

#### Experimental degradation of DNA

200 μL suspensions of 2.10^4^ J774 cells/mL, 2.10^5^ *M. smegmatis*/mL or 2.10^4^ *M. smegmatis*-infected J774 cells/mL were incubated in parallel at 90°C in a dry heat incubator (Techne Dri-Block®, Staffordshire, UK) for 1, 2, 4, 8, 12, 24 or 48 h. All experiments were conducted in triplicate. The cell suspensions not exposed to dry heat were included as negative controls. The DNA was extracted by adding 0.3 g of 106-μm glass beads (Sigma Aldrich, Steinheim, Germany) to 200 μL J774 cells or to *M. smegmatis* or *M. smegmatis*-infected J774 cells in 1.5 mL Eppendorf tubes. The cell suspensions were homogenized in a FastPrep-24 Instrument (MP Biomedicals Europe) 3 times for 20 s at 4 m/s and were then centrifuged at 16,045 x g for 3 min. DNA extraction was then performed using the QIAamp® DNA Mini kit (Qiagen, Hilden, Germany) following a modified protocol. Briefly, 200 μL ATL buffer and 20 μL proteinase K were added to each cell tube. The cell suspensions were vortexed for 15 s and then incubated at 56°C for 45 min. The 420-μL supernatant was transferred to a new Eppendorf tube and mixed with 200 μL absolute ethanol by vortexing for 15 s. The 620-μL mixture was transferred to a NucleoSpin column, and all following steps were conducted according to the QIAamp® DNA Mini Kit protocol. The resulting DNA was diluted with 60 μL AE buffer.

#### Real-time PCR measurements

**Figure 4:**
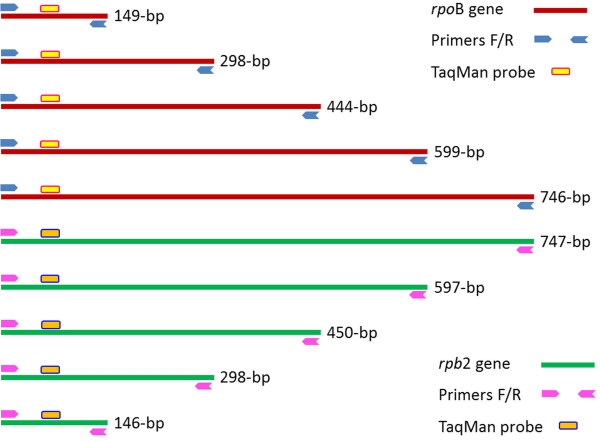
**Two primer systems with fluorescent probes specifically amplifying different fragments of the mycobacterial *rpo *B gene and its murine homolog *rpb *2.**

Two PCR primer systems were designed using the software Perlprimer version 1.1.6 [41]. The Rpb2.J774mur primer system specifically amplified sequences of different sizes (e.g., 146-bp, 298-bp, 450-bp, 597-bp and 747-bp) of the J774 *rpb*2 gene (gi|161898209|gb|EF536008.1|), and primer system RpoB.Msmeg amplified sequences of different sizes (149-bp, 298-bp, 444-bp, 599-bp and 746-bp) of the *M. smegmatis rpo*B gene (gi|34595742|gb|AY262735.1|). Two fluorescent probes, Rpb2.J774mur-TaqMan and RpoB.Msmeg-TaqMan, were designed to hybridize to the PCR-amplified *rpb*2 and *rpo*B fragments, respectively (Figure 4, Additional file 5: Table S5). Amplifications were performed in Stratagene^™^ MX3000P (Agilent Technologies Company, La Jolla, CA, USA) and CFX96^™^ Real-Time Systems (Bio-Rad, Singapore) using 10 μL reaction mixture (Quantitech, Qiagen), 2 μL sterile water, 2 μL Taqman® probe (Applied Biosystems, Villebon-sur-Yvette, France), 0.5 μL forward primer (10 μM), 0.5 μL reverse primer (10 μM) and 5 μL DNA by under the following PCR conditions: 15-min activation at 95°C and 40 cycles of 30-s denaturation at 95°C, 45-s hybridization at 62°C and 90-s elongation at 72°C. Ten negative controls (using DNA extracted from cells without 90°C dry heat exposure) were included in every batch. Additionally, ten blank controls (PCR-mix with sterile water instead of DNA) were also included. For each test sample, real-time PCR amplification was conducted in triplicate. Cut-offs were defined empirically; for each amplicon size, a test sample was considered as PCR-negative if its Ct value was greater than the Ct value of the negative control plus 12 (4,096 times of decreasing DNA concentration) or if its Ct value was nil. The Ct value of the test sample (Ctx) being 41 was delivered for no PCR-amplification or nil Ct values. PCR-detections of degraded DNA were confirmed as negative when all of three replicates were negative.

### Statistical analyses

The value of the negative control (Ct0) was used as baseline. For each amplicon size, the DNA degradation was calibrated by Ctx – Ct0. The ANOVA test was used to compare the means of (Ctx – Ct0) values.

## Competing interests

The authors declare that they have no competing interests.

## Authors’ contributions

TNH did the experiments, analyzed the data and drafted the manuscript. GA participated in the overall design and finalized the draft of the manuscript. MD designed the study, analyzed the data and finalized the draft of the manuscript. All the authors read and approved the final version of the manuscript.

## Supplementary Material

Additional file 1 **Table S1.**Average Ct values of *rpb*2 and *rpo*B amplified-fragments of J774 cells and *M. smegmatis.* Click here for file

Additional file 2 **Table S2.**ANOVA tests comparing means of (Ctx – Ct0) values. Click here for file

Additional file 3 **Table S3.**Average Ct values of *rpb*2 and *rpo*B amplified-fragments of *M. smegmatis*-infected J774 cells. Click here for file

Additional file 4 **Table S4.**References of mycobacterial DNA PCR-amplifications from ancient specimens (Figure 3) [1-24]. Click here for file

Additional file 5 **Table S5.**Primers and Taqman® probes used for real-time PCR quantification of J774 cell *rpb*2 and *M. smegmatis rpo*B genes. Click here for file
