# Supplementary material for: Heat degradation of eukaryotic and bacterial DNA: an experimental model for paleomicrobiology
Source: BMC Res Notes. 2012 Sep 25;5:528. doi: 10.1186/1756-0500-5-528 (PMC3532149; doi:10.1186/1756-0500-5-528)
Supplement: Additional file 1 — Table S1.Average Ct values of rpb2 and rpoB amplified-fragments of J774 cells and M. smegmatis. [file 1756-0500-5-528-S1.doc]

| Additional file 1: Table S1. Average Ct values of *rpb*2 and *rpo*B amplified-fragments of J774 cells and *M. smegmatis.* | | | | | | | | | | |
| --- | --- | --- | --- | --- | --- | --- | --- | --- | --- | --- |
|  | **J774 cells DNA – *rpb*2 gene** | | | | | ***M. smegmatis* DNA – *rpo*B gene** | | | | |
|  | ***146-bp*** | ***298-bp*** | ***450-bp*** | ***597-bp*** | ***747-bp*** | ***149-bp*** | ***298-bp*** | ***444-bp*** | ***599-bp*** | ***746-bp*** |
| **Controls** | 25.26 | 26.77 | 27.66 | 28.95 | 32.14 | 23.98 | 25.39 | 26.45 | 27.22 | 28.42 |
| **1 hour** | 28.93 | 30.89 | 32.33 | 33.25 | 37.41 | 22.59 | 24.23 | 23.81 | 25.23 | 26.49 |
| **2 hours** | 30.51 | 33.67 | 35.67 | 38.13 | 39.85 | 22.37 | 23.81 | 24.45 | 26.36 | 27.82 |
| **4 hours** | 31.89 | 35.01 | 35.99 | 39.00 | - | 22.11 | 23.68 | 24.14 | 25.35 | 26.89 |
| **8 hours** | 33.71 | 34.51 | 35.83 | 37.80 | - | 22.30 | 24.26 | 25.26 | 26.66 | 28.17 |
| **12 hours** | 33.42 | 34.77 | 35.78 | 38.60 | - | 24.24 | 26.42 | 26.77 | 28.35 | 29.77 |
| **24 hours** | 32.87 | 35.82 | 36.01 | 38.64 | - | 26.02 | 28.01 | 28.35 | 29.73 | 31.36 |
| **48 hours** | 33.01 | 35.83 | 37.53 | - | - | 27.80 | 30.31 | 30.03 | 31.90 | 34.56 |
