# Supplementary material for: Heat degradation of eukaryotic and bacterial DNA: an experimental model for paleomicrobiology
Source: BMC Res Notes. 2012 Sep 25;5:528. doi: 10.1186/1756-0500-5-528 (PMC3532149; doi:10.1186/1756-0500-5-528)
Supplement: Additional file 2 — Table S2.ANOVA tests comparing means of (Ctx – Ct0) values. [file 1756-0500-5-528-S2.doc]

| Additional file 2: Table S2. ANOVA tests comparing means of (Ctx – Ct0) values. | | | | |
| --- | --- | --- | --- | --- |
|  | J774 cells versus  *M. smegmatis* **(p-value)** | Infected J774 cells versus Intracellular  *M. smegmatis* **(p-value)** | J774 cells versus Infected J774 cells  **(p-value)** | *M. smegmatis* versus Intracellular  *M. smegmatis* **(p-value)** |
| 1 hour | 4.52 vs -1.33 **(0.000)** | 1.98 vs 2.00 **(0.914)** | 4.52 vs 1.98 **(0.000)** | -1.33 vs 2.00 **(0.000)** |
| 2 hours | 7.50 vs -0.81 **(0.000)** | 5.64 vs 2.95 **(0.000)** | 7.50 vs 5.64 **(0.001)** | -0.81 vs 2.95 **(0.000)** |
| 4 hours | 9.02 vs -1.36 **(0.000)** | 9.28 vs 3.67 **(0.000)** | 9.02 vs 9.28 **(0.772)** | -1.36 vs 3.67 **(0.000)** |
| 8 hours | 9.10 vs 0.51 **(0.000)** | 10.25 vs 4.07 **(0.000)** | 9.10 vs 10.25 **(0.214)** | 0.51 vs 4.07 **(0.000)** |
| 12 hours | 9.17 vs 2.29 **(0.000)** | 9.34 vs 6.64 **(0.028)** | 9.17 vs 9.34 **(0.836)** | 2.29 vs 6.64 **(0.000)** |
| 24 hours | 9.14 vs 3.81 **(0.000)** | 9.02 vs 6.26 **(0.151)** | 9.14 vs 9.02 **(0.925)** | 3.81 vs 6.26 **(0.109)** |
| 48 hours | 10.42 vs 5.89 **(0.000)** | 10.75 vs 6.82 **(0.005)** | 10.42 vs 10.75 **(0.743)** | 5.89 vs 6.82 **(0.416)** |
