# Supplementary material for: Heat degradation of eukaryotic and bacterial DNA: an experimental model for paleomicrobiology
Source: BMC Res Notes. 2012 Sep 25;5:528. doi: 10.1186/1756-0500-5-528 (PMC3532149; doi:10.1186/1756-0500-5-528)
Supplement: Additional file 3 — Table S3.Average Ct values of rpb2 and rpoB amplified-fragments of M. smegmatis-infected J774 cells. [file 1756-0500-5-528-S3.doc]

| Additional file 3: Table S3. Average Ct values of *rpb*2 and *rpo*B amplified-fragments of *M. smegmatis*-infected J774 cells. | | | | | | | | | | |
| --- | --- | --- | --- | --- | --- | --- | --- | --- | --- | --- |
|  | **J774 cells DNA – *rpb*2 gene** | | | | | ***M. smegmatis* DNA – *rpo*B gene** | | | | |
|  | ***146-bp*** | ***298-bp*** | ***450-bp*** | ***597-bp*** | ***747-bp*** | ***149-bp*** | ***298-bp*** | ***444-bp*** | ***599-bp*** | ***746-bp*** |
| **Controls** | 22.38 | 24.06 | 23.46 | 24.49 | 29.18 | 26.67 | 26.71 | 26.67 | 27.56 | 27.94 |
| **1 hour** | 24.36 | 26.01 | 25.64 | 26.72 | 30.09 | 28.60 | 28.61 | 28.79 | 29.52 | 29.92 |
| **2 hours** | 27.60 | 29.63 | 29.56 | 30.75 | 31.14 | 29.50 | 29.52 | 29.79 | 30.60 | 30.89 |
| **4 hours** | 30.34 | 32.48 | 33.86 | 34.99 | 35.54 | 30.12 | 30.38 | 30.58 | 31.47 | 31.55 |
| **8 hours** | 30.50 | 34.79 | 34.37 | 36.14 | 36.49 | 30.10 | 30.65 | 30.89 | 31.75 | 32.60 |
| **12 hours** | 30.40 | 33.71 | 33.60 | 34.64 | 36.06 | 32.08 | 32.49 | 30.09 | 32.29 | 32.84 |
| **24 hours** | 27.32 | 30.16 | 30.76 | 32.40 | 33.24 | 28.92 | 29.33 | 29.59 | 30.60 | 31.33 |
| **48 hours** | 30.02 | 34.56 | 34.88 | - | - | 31.43 | 32.22 | 31.36 | 34.96 | 34.15 |
