# Supplementary material for: Heat degradation of eukaryotic and bacterial DNA: an experimental model for paleomicrobiology
Source: BMC Res Notes. 2012 Sep 25;5:528. doi: 10.1186/1756-0500-5-528 (PMC3532149; doi:10.1186/1756-0500-5-528)
Supplement: Additional file 4 — Table S4.References of mycobacterial DNA PCR-amplifications from ancient specimens (Figure 3) [1-24]. [file 1756-0500-5-528-S4.doc]

Additional file 4: Table S4. References of mycobacterial DNA PCR-amplifications from ancient specimens (Figure 3) [1-24].

1. Donoghue HD, Spigelman M, Zias J, Gernaey-Child AM, Minnikin DE: ***Mycobacterium tuberculosis* complex DNA in calcified pleura from remains 1400 years old**. *Lett Appl Microbiol* 1998, **27:** 265-269.

2. Donoghue HD, Holton J, Spigelman M: **PCR primers that can detect low levels of *Mycobacterium leprae* DNA**. *J Med Microbiol* 2001, **50:** 177-182.

3. Donoghue HD, Marcsik A, Matheson C, Vernon K, Nuorala E, Molto JE, Greenblatt CL, Spigelman M: **Co-infection of *Mycobacterium tuberculosis* and *Mycobacterium leprae* in human archaeological samples: a possible explanation for the historical decline of leprosy**. *Proc Biol Sci* 2005, **272:** 389-394.

4. Donoghue HD, Lee OY, Minnikin DE, Besra GS, Taylor JH, Spigelman M: **Tuberculosis in Dr Granville's mummy: a molecular re-examination of the earliest known Egyptian mummy to be scientifically examined and given a medical diagnosis**. *Proc Biol Sci* 2010, **277:** 51-56.

5. Fletcher HA, Donoghue HD, Taylor GM, van der Zanden AG, Spigelman M: **Molecular analysis of *Mycobacterium tuberculosis* DNA from a family of 18th century Hungarians**. *Microbiology* 2003, **149:** 143-151.

6. Haas CJ, Zink A, Molnar E, Szeimies U, Reischl U, Marcsik A, Ardagna Y, Dutour O, Palfi G, Nerlich AG: **Molecular evidence for different stages of tuberculosis in ancient bone samples from Hungary**. *Am J Phys Anthropol* 2000, **113:** 293-304.

7. Haas CJ, Zink A, Palfi G, Szeimies U, Nerlich AG: **Detection of leprosy in ancient human skeletal remains by molecular identification of *Mycobacterium leprae***. *Am J Clin Pathol* 2000, **114:** 428-436.

8. Hershkovitz I, Donoghue HD, Minnikin DE, Besra GS, Lee OY, Gernaey AM, Galili E, Eshed V, Greenblatt CL, Lemma E, Bar-Gal GK, Spigelman M: **Detection and molecular characterization of 9,000-year-old *Mycobacterium tuberculosis* from a Neolithic settlement in the Eastern Mediterranean**. *PLoS One* 2008, **3:** e3426.

9. Konomi N, Lebwohl E, Mowbray K, Tattersall I, Zhang D: **Detection of mycobacterial DNA in Andean mummies**. *J Clin Microbiol* 2002, **40:** 4738-4740.

10. Likovsky J, Urbanova M, Hajek M, Cerny V, Cech P: **Two cases of leprosy from Zatec (Bohemia), dated to the turn of the 12th century and confirmed by DNA analysis for *Mycobacterium leprae***. *J Archaeol Sci* 2006, **33:** 1276-1283.

11. Matheson CD, Vernon KK, Lahti A, Fratpietro R, Spigelman M, Gibson S, Greenblatt CL, Donoghue HD, Zissu B: **Molecular exploration of the first-century Tomb of the Shroud in Akeldama, Jerusalem**. *PLoS One* 2009, **4:** e8319.

12. Mays S, Taylor GM, Legge AJ, Young DB, Turner-Walker G: **Paleopathological and biomolecular study of tuberculosis in a medieval skeletal collection from England**. *Am J Phys Anthropol* 2001, **114:** 298-311.

13. Montiel R, Garcia C, Canadas MP, Isidro A, Guijo JM, Malgosa A: **DNA sequences of *Mycobacterium leprae* recovered from ancient bones**. *FEMS Microbiol Lett* 2003, **226:** 413-414.

14. Rothschild BM, Martin LD, Lev G, Bercovier H, Bar-Gal GK, Greenblatt C, Donoghue H, Spigelman M, Brittain D: ***Mycobacterium tuberculosis* complex DNA from an extinct bison dated 17,000 years before the present**. *Clin Infect Dis* 2001, **33:** 305-311.

15. Salo WL, Aufderheide AC, Buikstra J, Holcomb TA: **Identification of *Mycobacterium tuberculosis* DNA in a pre-Columbian Peruvian mummy**. *Proc Natl Acad Sci U S A* 1994, **91:** 2091-2094.

16. Suzuki K, Takigawa W, Tanigawa K, Nakamura K, Ishido Y, Kawashima A, Wu H, Akama T, Sue M, Yoshihara A, Mori S, Ishii N: **Detection of *Mycobacterium leprae* DNA from archaeological skeletal remains in Japan using whole genome amplification and polymerase chain reaction**. *PLoS One* 2010, **5:** e12422.

17. Taylor GM, Goyal M, Legge AJ, Shaw RJ, Young D: **Genotypic analysis of *Mycobacterium tuberculosis* from medieval human remains**. *Microbiology* 1999, **145 ( Pt 4):** 899-904.

18. Taylor GM, Widdison S, Brown IN, Young D: **A mediaeval case of lepromatos leprosy from 13-14th century Orkney, Scotland**. *J Archaeol Sci* 2000, **27:** 1133-1138.

19. Taylor GM, Watson CL, Bouwman AS, Lockwood DNJ, Mays SA: **Variable nucleotide tandem repeat (VNTR) typing of two palaeopathological cases of lepromatous leprosy from Mediaeval England**. *J Archaeol Sci* 2006, **33:** 1569-1579.

20. Taylor GM, Blau S, Mays S, Monot M, Lee OYC, Minnikin DE, Besra GS, Cole ST, Rutland P: ***Mycobacterium leprae* genotype amplified from an archaeological case of lepromatous leprosy in Central Asia**. *J Archaeol Sci* 2009, **36:** 2408-2414.

21. Taylor GM, Donoghue HD: **Multiple loci variable number tandem repeat (VNTR) analysis (MLVA) of *Mycobacterium leprae* isolates amplified from European archaeological human remains with lepromatous leprosy**. *Microbes Infect* 2011, **13:** 923-929.

22. Watson CL, Lockwood DN: **Single nucleotide polymorphism analysis of European archaeological *M. leprae* DNA**. *PLoS One* 2009, **4:** e7547.

23. Zink A, Haas CJ, Reischl U, Szeimies U, Nerlich AG: **Molecular analysis of skeletal tuberculosis in an ancient Egyptian population**. *J Med Microbiol* 2001, **50:** 355-366.

24. Zink AR, Sola C, Reischl U, Grabner W, Rastogi N, Wolf H, Nerlich AG: **Characterization of *Mycobacterium tuberculosis* complex DNAs from Egyptian mummies by spoligotyping**. *J Clin Microbiol* 2003, **41:** 359-367.
