# Supplementary material for: Heat degradation of eukaryotic and bacterial DNA: an experimental model for paleomicrobiology
Source: BMC Res Notes. 2012 Sep 25;5:528. doi: 10.1186/1756-0500-5-528 (PMC3532149; doi:10.1186/1756-0500-5-528)
Supplement: Additional file 5 — Table S5.Primers and Taqman® probes used for real-time PCR quantification of J774 cell rpb2 and M. smegmatis rpoB genes. [file 1756-0500-5-528-S5.doc]

| Additional file 5: Table S5. Primers and Taqman® probes used for real-time PCR quantification of J774 cell *rpb*2 and *M. smegmatis* *rpo*B genes. | | | | |
| --- | --- | --- | --- | --- |
| ***Systems primers*** | ***Name*** | ***Sequence (5’ – 3’)*** | ***Amplicon*** | ***Tm*** |
| Rpb2.J774mur | Rpb2.J774mur-F | CTATAACCTGAATGTAGCAAGC |  | 62°C |
|  | Rpb2.J774mur-R1 | CCCAATGAGGTGCTAGACTC | 146-bp |  |
|  | Rpb2.J774mur-R2 | AATATTAACCAAGTTTAGAAACGC | 298-bp |  |
|  | Rpb2.J774mur-R3 | CCTAGCACGAGAATGAATTTTG | 450-bp |  |
|  | Rpb2.J774mur-R4 | CATCACTCGCCGCCTCTAC | 597-bp |  |
|  | Rpb2.J774mur-R5 | TATATCCCAAGTTTTATGAAGGG | 747-bp |  |
|  | Rpb2.J774mur-TaqMan | 6FAM-TGCAAGATGTCATAGGCATACAGC-TAMRA |  |  |
| RpoB.Msmeg | RpoB.Msmeg-F | TCTCCGAGATCATGATGGGC |  | 62°C |
|  | RpoB.Msmeg-R1 | CTCCTTGAAGAACAGGTTCTC | 149-bp |  |
|  | RpoB.Msmeg-R2 | GTCTGACCCTCGTGCAGAC | 298-bp |  |
|  | RpoB.Msmeg-R3 | CACGCACGACACGCTCCAT | 444-bp |  |
|  | RpoB.Msmeg-R4 | ACGCTTGTGGGTCAGACCC | 599-bp |  |
|  | RpoB.Msmeg-R5 | CACCGACAGCGAACCGATC | 746-bp |  |
|  | RpoB.Msmeg-TaqMan | 6 FAM-ACGAGGCCCTGCTCGACATCTA-TAMRA |  |  |
